# Disrupted Hierarchical Functional Brain Organization in Affective and Psychotic Disorders: Insights from Functional Brain Gradients

**DOI:** 10.21203/rs.3.rs-6287335/v1

**Published:** 2025-04-17

**Authors:** Joseph Kambeitz, Hannah Hacker, Linnea Hoheisel, Madalina Buciuman, Annkathrin Böke, Theresa Lichtenstein, Marlene Rosen, Shalaila Haas, Anne Ruef, Dominic Dwyer, Paolo Brambilla, Carolina Bonivento, Rachel Upthegrove, Stephen Wood, Stefan Borgwardt, Eva Meisenzahl, Stephan Ruhrmann, Raimo Salokangas, Udo Dannlowski, Nikolaos Koutsouleris, Lana Kambeitz-Ilankovic, Rebekka Lencer

**Affiliations:** Faculty of Medicine and University Hospital University of Cologne, Cologne; Icahn School of Medicine at Mount Sinai; LMU; Università di Milano; University of Melbourne; University of Lübeck; HHU Düsseldorf; Faculty of Medicine and University Hospital, University of Cologne, Cologne; University of Turku; University of Münster; Ludwig-Maximilians-University; University of Cologne, Faculty of Medicine and University Hospital

## Abstract

Patients with psychosis and depression show widespread alterations in brain resting-state functional connectivity (rs-FC), affecting both sensory and higher-order brain regions. In this study, we investigate disruptions in the hierarchical organization of brain functional networks in patients with psychotic and affective disorders. We derived functional brain gradients, low dimensional representations of rs-FC that capture cortical hierarchy, in a large patient sample including clinical high-risk for psychosis (CHR-P) patients, recent-onset psychosis (ROP) patients, recent-onset depression (ROD) patients, and healthy controls (HC). We examined regional alterations, network-level alterations and functional differentiation and their relationship to clinical symptoms. In addition, we linked case-control differences to receptor expression maps to explore underlying neurobiological mechanisms. All patient groups exhibited alterations in the visual-to-sensorimotor gradient, while only ROP patients showed alterations in the association-to-sensory gradient. CHR-P and ROP patients exhibited lower values in the ventral attention network. Additionally, patients combined showed higher values in the somatomotor network, a reduced gradient range and altered between-network dispersion. ROD showed reduced within-network dispersion in the attentional networks and a reduced range. Correlational analysis revealed weak associations of gradient measures with functioning, visual dysfunctions and cognition. Furthermore case-control differences showed associations to receptor expression maps, suggesting the involvement of neurotransmitter systems in these disruptions. Our findings reveal transdiagnostic and disease-specific alterations of hierarchical brain organization. These alterations indicate deficits in functional integration across psychiatric diseases, highlighting the role of attentional and sensory networks in disease processes.

## Introduction

Psychotic and depressive disorders represent a major burden for patients and society due to their high prevalence and impact on psychosocial functioning (1). Affected patients experience a wider range of specific symptoms, including those in affective, attentional and sensory domains. Despite the distinct symptoms associated with each disorder, there exists a wide heterogeneity within disorders (2). Furthermore, depressive and psychotic syndromes frequently co-occur (3). Beyond shared clinical symptoms, increasing evidence suggests overlapping neurobiological abnormalities (4–7), including alterations in brain functional connectivity (8). These clinical and neurobiological overlaps raise the question whether these diseases share similar underlying neural alterations and underline the need to identify shared disease mechanisms.

Previous research has shown that resting-state functional connectivity (rs-FC) is altered in patients with depression, in patients with schizophrenia and even in individuals with clinical high-risk for psychosis (CHR-P) (9–11). Observed alterations are present in multiple brain networks (11–16) including sensory processing areas and areas relevant to higher-order processes such as the frontoparietal network (FPN), the default mode network (DMN) and the dorsal attention network (DAN) (15, 16).

While some studies point to regional alterations of rs-FC in psychosis and depression, there is increasing evidence indicating changes in overall *network structure* (17,18).

This observation has motivated novel analysis approaches focusing on the *hierarchical* organization of brain rs-FC in depression and psychosis. As an example the hierarchical organization of the cortex can be characterized through low dimensional representations of rs-FC (19). These so-called functional brain gradients position regions along axes of variation in their coactivation profiles in rs-FC. Analyzing gradients can provide insights about the position of regions along the spectrum of information-processing (20). Typically, the first gradient extends from unimodal sensory areas to trans-modal association areas like the default mode network (DMN), while the second gradient extends from visual to sensorimotor areas (19). These gradients have been found to correspond to myelination and other measures like macrostructural features (19), cortical thickness and receptor expression (21). It has been suggested that the hierarchical organization of brain gradients corresponds to the hierarchy present in cognitive processes (22). Following this hypothesis, the processing of sensory information into abstract representations corresponds to the first gradient which spans from unimodal to transmodal cortices, while the second gradient separates unimodal processing areas (19). Alterations in gradients would point towards disturbances in this hierarchical stream of processing. Recent studies have shown various alterations in brain gradient scores in patients with depression or psychosis indicating changes in functional integration and segregation compared to healthy participants (23–26). Some alterations are also detectable in individuals with a high vulnerability to depression (27).

In patients with schizophrenia studies report alterations in the visual-to-sensorimotor gradient (23), both the visual-to-sensorimotor and the sensorimotor-to-association gradient (28) or increased dispersion in multiple brain networks indicating that the differentiation between networks in schizophrenia is blurred (23). Moreover, other studies show alterations in cortical hierarchy in earlier disease stages such as recent-onset psychosis (ROP) (26). Some initial studies report associations between symptoms and gradient alterations in patients with schizophrenia and depression (23, 25). Until today it is not known if alterations in hierarchical organization are already present in individuals with high-risk for psychosis.

In the present study, we aim to investigate the alterations in cortical hierarchy in early phases of affective and psychotic disorders as well as individuals at clinical high-risk for psychosis. Specifically, we focus on the shared and diagnosis-specific alterations of cortical hierarchical organization and investigate associations with clinical and cognitive features. As functional gradients have been related to neurotransmitter receptor expression before, we additionally examined the relationship between possible alterations and receptor maps.

## Material and Methods

### Data

Here, data from the PRONIA study (Personalized Prognostic tools for early psychosis management, http://www.pronia.eu/) (29) was analyzed. This study has been conducted across 10 sites in Europe and includes HC subjects (*n* = 393), patients with ROP (*n* = 255), patients with recent-onset depression (ROD) (*n* = 229), and patients with CHR-P (*n* = 247), who underwent resting state functional magnetic resonance imaging (rs-fMRI). Inclusion and exclusion criteria can be found in the Supplementary Methods.

Prior to study inclusion all participants provided their written informed consent themselves or in case of minor participants, informed consent was provided by their guardians. At each location, the study was approved by the local research ethics committees and registered at the German Clinical Trials Register (DRKS00005042).

#### Clinical and Neuropsychological Assessment

The Schizophrenia Proneness Instrument (SPI-A) (30) and Structured Interview for Psychosis-Risk Syndromes (31) were used to assess CHR-P status. SPI-A items related to visual dysfunctions were used to calculate a visual dysfunctions (VisDys) score as described before (14). Further positive, negative and general psychotic symptoms were assessed by the Positive and Negative Syndrome Scale (PANSS) (32). Depressive symptoms were assessed via self-report using the Beck’s Depression Inventory – II (33). The Global Functioning: Role Scale (GF-Role) and Global Functioning: Social Scale (GF-Social) (34) were used to assess global role and social functioning. Furthermore the Global Assessment of Functioning (GAF) (35) was administered.

Five cognitive domains were measured by seven tests. The domains included social cognition, working memory, speed of processing, verbal learning and attention, building together the global cognition score. The tests used were Diagnostic Analysis of Non-Verbal Accuracy (36), semantic verbal fluency (37), Rey Auditory Verbal Learning Test (38), Trail-Making Test Part A (39), Continuous Performance Test - Identical Pairs (40), Wechsler Memory Scale: spatial span subtest (forward and backward) and the Digit-Symbol-Substitution Task (41). More information on the computation of the scores can be found in the Supplementary Methods.

For analyses regarding the association to clinical variables, participants with more than 30 percent missing data points in the clinical scales were excluded. For participants with fewer missing values the values were imputed by the median of the corresponding group.

#### MRI acquisition and preprocessing

T1 reference images were acquired using a multi-echo MPRAGE sequence. At all sites, rs-fMRI scans were acquired using echo planar imaging sequences with 200 volumes and a repetition time of 3 seconds, resulting in a duration of 603 seconds. The participants were instructed to keep their eyes open throughout the scan. As acquisition parameters differed between acquisition sites, details of the acquisition sites can be found in the Supplementary Tables 1 and 2. The data was preprocessed with a pipeline developed by the PRONIA consortium (42). Details can be found in the Supplementary Methods.

#### Functional Gradient Analysis

**Figure 1:**
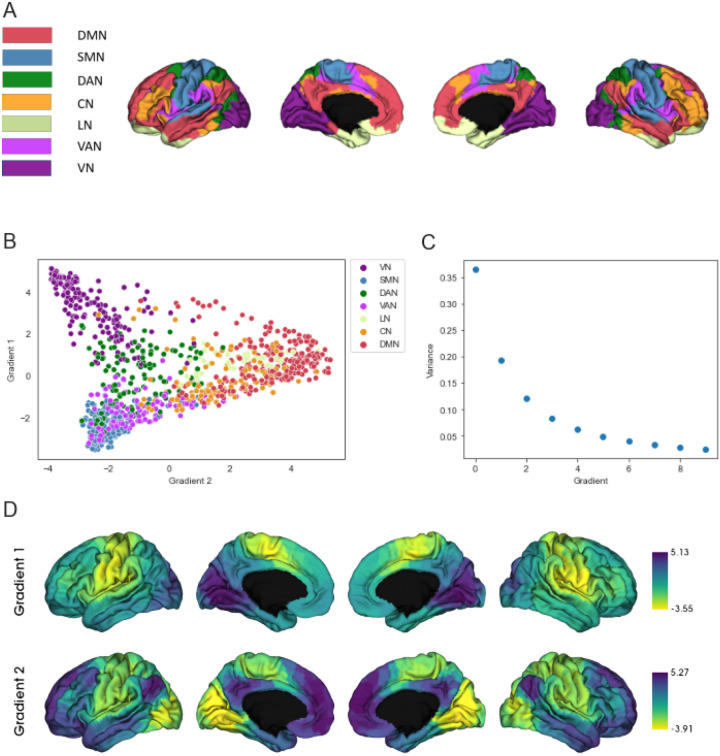
Gradient structure across groups. (A) Schaefer 7 network parcellation. (B) Average gradient scores in the two-dimensional gradient space. (C) Mean variance explained by gradients. (D) Spatial topography of first and second gradient. CN = control network; DAN = dorsal attention network; DMN = default mode network; LN = limbic network; SMN = somatomotor network; VAN = ventral attention network (salience); VN = visual network.

In accordance with previous studies (23, 43), we used the Schaefer parcellation (44) with 1 000 parcels, which includes annotations of the 7 Yeo networks (45) (Fig. 1), to derive a functional connectivity matrix. This allowed us to investigate network specific alterations in the DAN, the FPN, the DMN, the LN, the VN, the somatomotor network (SMN) and the ventral attention network (VAN). Pearson correlation was used to compute a FC matrix for every participant, which was then standardized using the Fisher z-transformation. The gradients were derived with the Python implementation of the BrainSpace toolbox (https://github.com/MICA-MNI/BrainSpace; Vos De Wael et al., 2020), using the FC matrix as the input. In line with previous research (23, 47), the FC matrices were thresholded to retain the strongest 10% of connections of each region. From this matrix, an affinity matrix was computed using cosine similarity and decomposed into a set of principal eigenvectors using diffusion mapping as a dimensionality reduction method. These eigenvectors serve to describe the underlying gradients (46). Individual gradients were aligned to a reference gradient derived from the mean FC from the healthy controls in our study sample via Procrustes rotation (48). The reference gradients are reported in the Supplementary Fig. 2. In order to account for differences in MRI scanners between sites, we employed ComBat (49) on the gradient scores and derived metrics. Details can be found in the Supplementary Methods. Differences in scores of the first two gradients between patient groups and healthy controls were explored regionally using the surface-based linear model (SLM) from the BrainStat toolbox (50), with age, sex and mean framewise displacement as covariates. P-values were corrected for multiple comparisons by the false discovery rate (FDR) correction (51). Group differences in mean network values were analyzed using Mann-Whitney-U Tests, after confound regression of age, sex and mean framewise displacement, as data were not normally distributed. Also here, we applied FDR to correct for multiple comparisons. We assessed the range of gradient scores as a measure for functional differentiation by subtracting the minimum from the maximum gradient value of each subject. In a next step, we investigated network-specific functional differentiation by deriving measures of within- and between-network dispersion (47) in the two-dimensional gradient space. For this, the central region of each network was computed as the median of all regions in that network. Each region is represented by two coordinates, with the first coordinate indicating the value of that region on the first gradient and the second coordinate representing the value of this region on the second gradient. Within-Network dispersion was calculated as the mean Euclidean distance between this central region and all other regions belonging to that network. Between-Network dispersion was calculated as the Euclidean distance between the central regions of different networks, leading to 7 within-network dispersion scores and 21 between-network dispersion scores per participant. Group differences in these measures were also evaluated using Mann-Whitney-U-test and FDR-correction was again used to correct for multiple comparisons.

We used correlation analyses to explore associations between gradient dispersion metrics and network gradient values and clinical variables.

We furthermore evaluated associations between t-maps of HC and patients and open available receptor expression maps from PET scans using the neuromaps toolbox (52). We used all available receptor maps covering different receptors of nine neurotransmitter systems including receptors/transporters for acetylcholine, dopamine, cannabinoid, opioid, glutamate, serotonin, GABA, norepinephrene and histamine (see Supplementary Table 3 for more details). Maps for the same receptor types were averaged (see Supplementary Fig. 3 for correlations). Significance was tested based on 10 000 spin permutations (53) to account for spatial autocorrelation. To furthermore account for multiple comparisons, results were then FDR-corrected.

## Results

### Demographic and clinical characteristics

Of the 1 124 participants that underwent rs-fMRI, 25 participants were excluded because of motion in the MRI or signal loss leading to missing values in the FC matrix. Furthermore, we excluded 22 participants because of missing values for sex or age. We also decided to exclude participants from Bari because in total only six ROD and ROP patients were recruited there, making it impossible to correct for site effects. This led to a sample size of n = 1 071 for group analyses. For analyses regarding the association to clinical variables, 18 additional participants were excluded due to missing data (Supplementary Fig. 1).

We found significant differences in age, sex, education years and chlorpromazine equivalent (CPZE) distribution between the diagnostic groups (Table 1). For the analysis of association between gradients and clinical symptoms, patient groups showed significant differences in sex, age and all analyzed clinical variables (Table 2).

#### Functional gradient differences between HC and patients

We found the first gradient in our data to span sensorimotor and visual areas and the second gradient to span the DMN and sensorimotor areas (Fig. 1). Neither for gradient 1 (*F*(3, 1 067) = 1.393, *p* = .243) nor for gradient 2 (*F*(3, 1 067) = 0.688, *p* = .559) did the explained variances differ between groups, allowing us to compare gradients between groups and showing that the same gradients can be found in patients and HC. The visual-to-sensorimotor gradient explained on average 36.25% (*SD* = 7.90) of variance for HC, 36.32% (*SD* = 8.41) for CHR-P, 37.41% (*SD* = 8.26) for ROP and 36.09% (*SD* = 7.63) for ROD. The sensory-to-association gradient explained on average 19.50% (*SD* = 4.26) of variance for HC, 19.55% (*SD* = 3.93) for CHR-P, 19.27% (*SD* = 4.18) for ROP and 19.09% (*SD* = 3.60) for ROD.

**Figure 2:**
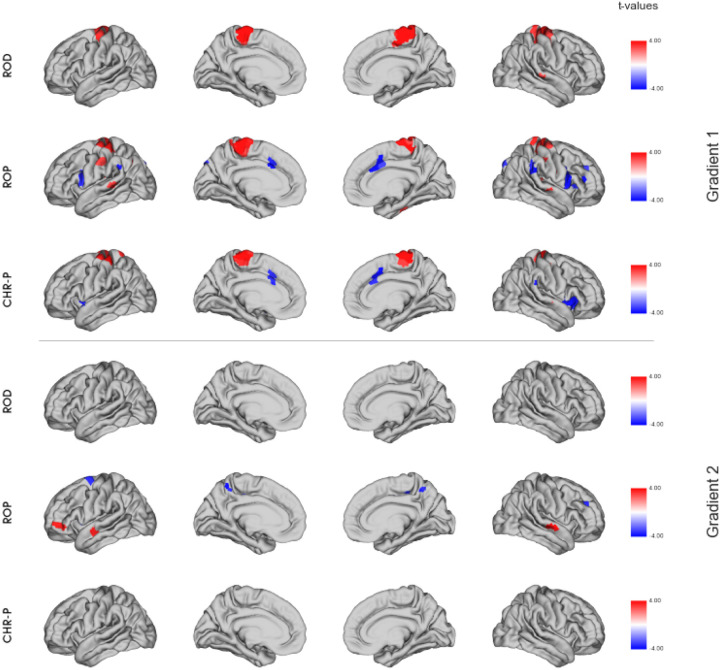
*t*-values of regions that significantly differ in their gradient value between HC and patient groups after FDR-correction with *p*_FDR_ < .05; models were controlled for age, sex and mean framewise displacement; higher/lower values in patient groups are presented in red/blue. CHR-P = clinical high-risk; ROD = recent-onset depression, ROP = recent-onset psychosis.

All three patient groups showed higher scores in the SMN in the visual-to-sensorimotor gradient compared to HC. For ROP patients, significant differences also appeared in regions belonging to all other networks, but most prominent were regions of the VAN, with ROP patients showing lower values than HC. CHR-P patients also exhibited significantly lower values in regions of the VAN and additionally showed some significant differences in a few regions of the CN, LN and DMN. ROD patients showed a few single significant regions in the CN, DAN and VAN. Alterations in the association-to-sensory gradient were less pronounced. ROD patients only showed significant differences in one region, belonging to the CN. ROP patients showed a few significant regions in the DAN, CN, DMN and SMN. CHR-P patients showed no significant differences in the association-to-sensory gradient. Statistical differences in the gradients are displayed on the cortical surface in Fig. 2.

**Figure 3:**
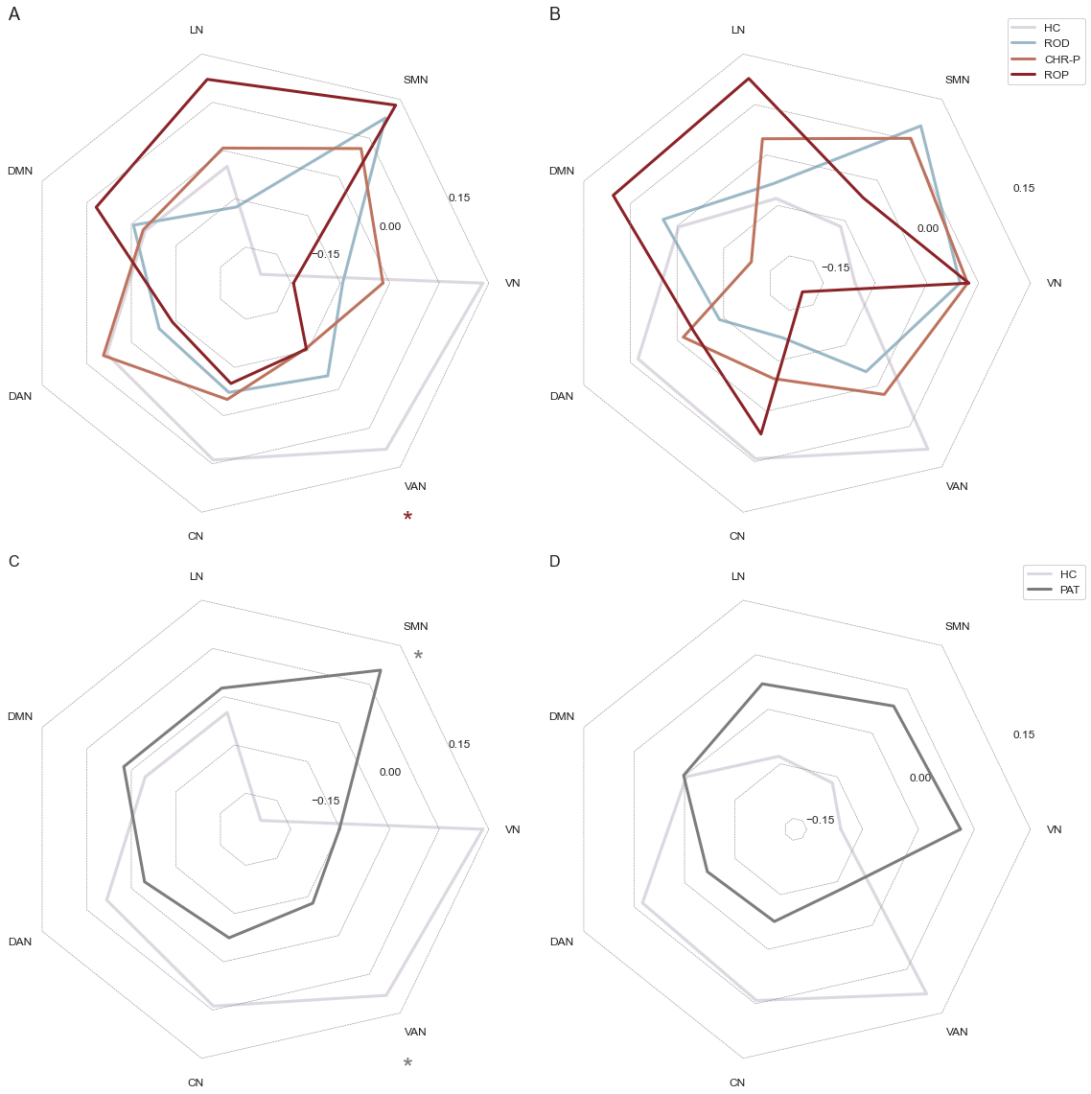
Differences between patient groups and healthy controls in mean network values. Gradient values are displayed after regressing out age, sex and mean framewise displacement. Colors of stars indicate which groups differ from HC after FDR-correction with *p*_FDR_ < .05. (A) Gradient 1 for HC vs. patient groups. (B) Gradient 2 for HC vs. patient groups. (C) Gradient 1 for HC vs. patients combined. (D) Gradient 2 for HC vs. patients combined. CHR-P = clinical high-risk; CN = control network; DAN = dorsal attention network; DMN = default mode network; HC = healthy controls; LN = limbic network; Pat = patient combined; ROD = recent-onset depression; ROP = recent-onset psychosis; SMN = somatomotor network; VAN = ventral attention network; VN = visual network.

The analysis of mean network values confirmed the findings of the regional analysis, which indicated that the focus of alterations was on the first gradient. For the visual-to-sensorimotor gradient we found significantly lower scores in the VAN for ROP patients (*U* = 52 930, *p*_*FDR*_ = .022, η^2^ = .0131) and patients in general (*U* = 149 052, *p*_*FDR*_ = .004, η^2^ = .0117) compared to HC. CHR-P patients also showed lower values in the VAN, but this difference did not survive FDR-correction (*U* = 51 736, *p*_*FDR*_ = .052, η^2^ = .0134). Furthermore, we found higher scores in the SMN when comparing all patients to HC (*U* = 113 120, *p*_*FDR*_ = .008, η^2^ = .0168). We did not find any significant differences regarding the association-to-sensorimotor gradient on the network-level after FDR-correction (Fig. 3).

#### Functional differentiation

Overall, patients showed a smaller range of the visual-to-sensorimotor gradient compared to HC (*U* = 143 701, *p*_FDR_ = .021, η^2^ = .0041). This effect was also present when comparing only ROD to HC (*U* = 45 508, *p*_FDR_ = .017, η^2^ = .0083). There were no differences between any patient group and HC in the sensory-to-association gradient.

**Figure 4:**
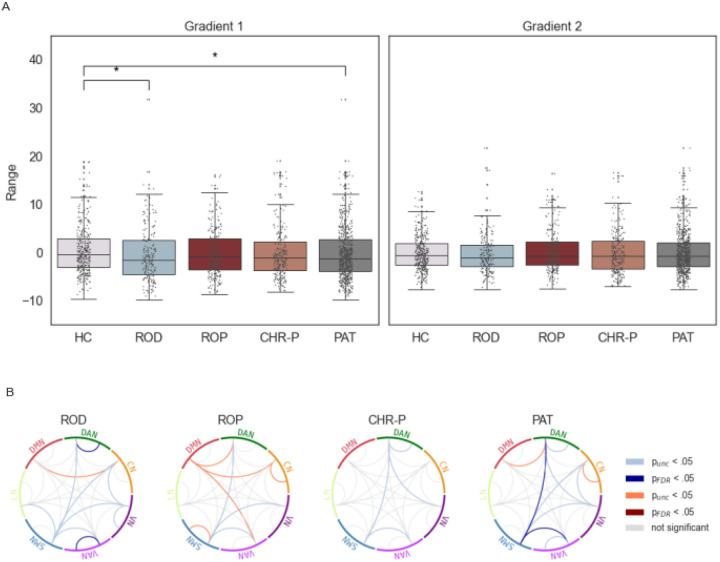
Functional Differentiation. (A) Range of gradients. Whiskers indicate interquartile range. Asterisks indicate significant differences after FDR-correction; * *p*_FDR_ < .05. (B) Between-group differences in within- and between-network dispersion. Grey lines indicate non-significant differences. Red/dark blue lines indicate significantly higher/lower values for the respective patient group with *p*_FDR_ < .05 after FDR-correction. Orange/light blue lines indicate significantly higher/lower values for the respective patient group before FDR-correction. CHR-P = clinical high-risk; CN = control network; DAN = dorsal attention network; DMN = default-mode network; HC = healthy control; LN = limbic network; PAT = patients combined; ROD = recent-onset depression; ROP = recent-onset psychosis; SMN = somatomotor network; VAN = ventral attention network; VN = visual network.

Analyses of the network dispersion revealed significant differences in the within-network dispersion only for ROD patients in the DAN (*U* = 46 729, *p*_FDR_ = .014, η^2^ = .0119) and VAN (*U* = 46 296, *p*_FDR_ = .030, η^2^ = .0112), with ROD patients exhibiting lower scores than HC. Analysis of between-network dispersion showed lower scores for the dispersion in patients between SMN and VAN (*U* = 148 271, *p*_FDR_ = .022, η^2^ = .0144) and between SMN and DAN (*U* = 147 291, *p*_FDR_ = .047, η^2^ = .0123) as compared to HC after FDR correction. Differences in separate patient group analyses did not survive FDR-correction but indicated numerically lower scores in all patient groups as compared to HC (Fig. 4).

### Association with clinical symptoms

**Figure 5:**
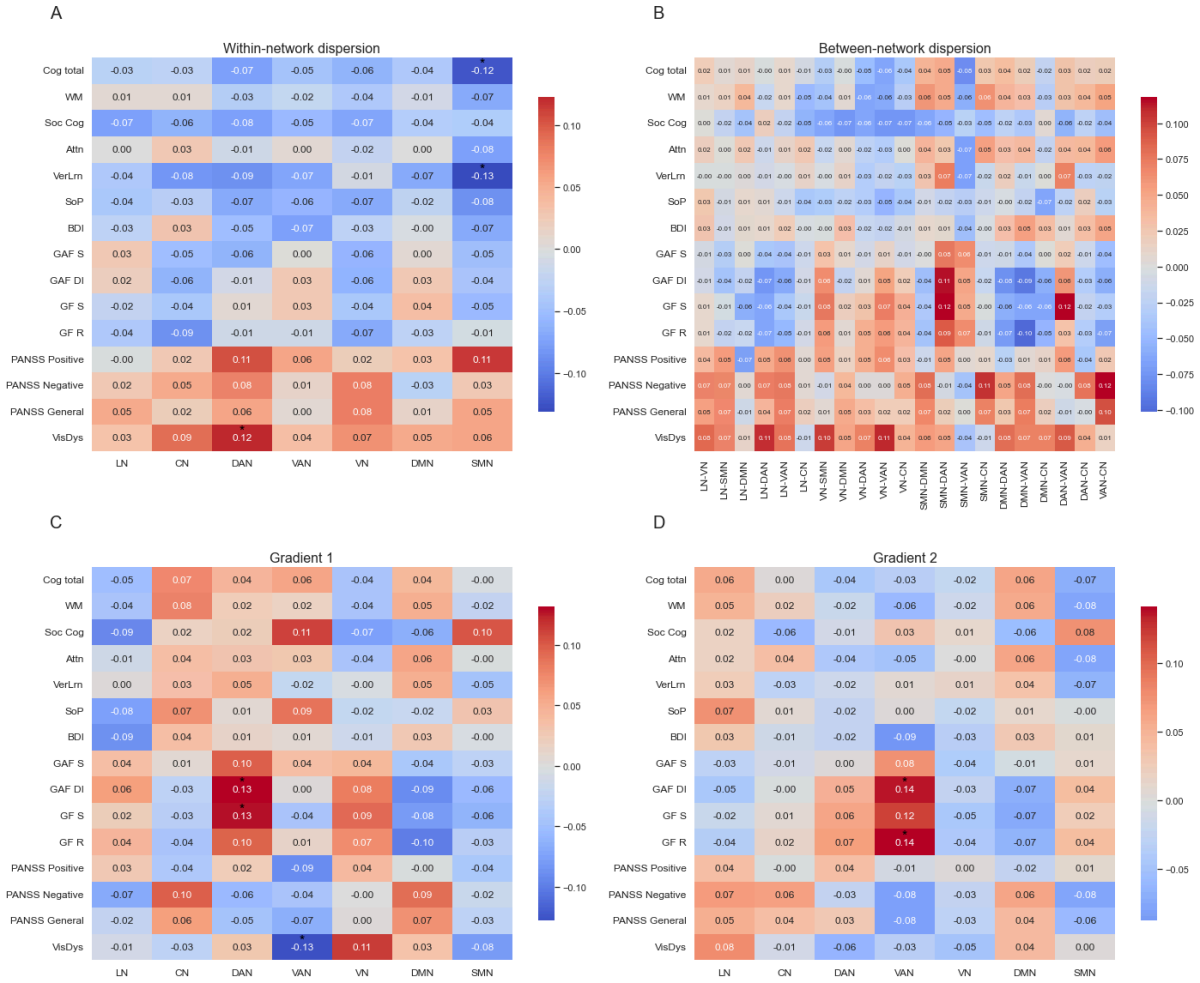
Association of clinical variables with gradient measures. Asterisks indicate significant correlations after FDR-correction; * *p*_FDR_ < .05. (A) Correlations of within-network dispersion with clinical variables. (B) Correlations of between-network dispersion with clinical variables. (C) Correlations of mean-network values of gradient 1 with clinical variables. (D) Correlations of mean-network values of gradient 2 with clinical variables. Attn = attention; BDI = Becks Depression Inventory; CN = control network; Cog total = cognition total score; DAN = dorsal attention network; DMN = default-mode network; GAF DI = Global Assessment of Functioning Disability; GAF S = Global Assessment of Functioning Symptoms; GF R = Global Functioning Role Scale; GF S = Global Functioning Social Scale; LN = limbic network; Soc Cog = social cognition; SoP = speed of processing; SMN = somatomotor network; VAN = ventral attention network; VerLrn = verbal learning; VisDys = visual dysfunctions; VN = visual network; WM = working memory.

We found small correlations between clinical measures of functioning and mean network values of the visual-to-sensorimotor gradient and the sensory-to-association in the attentional networks after FDR-correction. Mean values of the DAN of the visual-to-sensorimotor gradient correlated significantly with GF-S and GAF-DI scores and the mean values of the VAN of the sensory-to-association gradient correlated significantly with GF-R and GAF-DI scores. Moreover, visual dysfunctions were associated with mean values of the VAN of the visual-to-sensorimotor gradient and within-network dispersion of the DAN (Fig. 5). Within-network dispersion of the SMN was associated with verbal learning and total cognition scores. We repeated these analyses for groups separately, after which no associations remained significant (Supplementary Fig. 6–8).

#### Association with receptor expression

**Figure 6:**
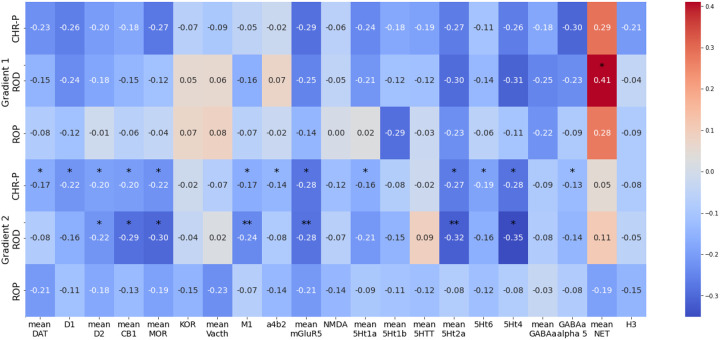
Association of receptor maps with case-control differences in gradients. Asterisks indicate significant correlations after FDR-correction; * *p*_spin-FDR_ < .05; ** *p*_spin-FDR_ < .01. CHR-P = clinical high-risk; ROD = recent-onset depression; ROP = recent-onset psychosis.

We identified spatial concordances between the gradient t-maps of patient groups and receptor expression maps. Most pronounced were associations for ROD patients between serotonin receptors and variations in the sensory-to-association gradient. CHR-P patients showed associations with the same receptor maps as ROD patients, but also to additional receptor maps. No significant associations were identified for ROP patients. The only significant association for the visual-to-sensorimotor gradient was found for ROD patients with NET (Fig. 6).

#### Sensitivity Analyses

We correlated the significant alterations for ROP patients in the VAN with daily CPZE to check if effects could be caused by medication, but did not find a significant correlation (Supplementary Fig. 4).

We also tested the influence of choosing a different threshold before computing the affinity matrix, retaining the strongest 20% of connections. That switched the order of the gradients, but there was still a high correspondence (r = .925) between the visual-to-sensorimotor gradients and between the sensory-to-association gradients (Supplementary Fig. 5).

## Discussion

In this study we investigated disease-specific and transdiagnostic alterations of hierarchical brain organization in functional brain gradients of rs-FC and their association to clinical symptoms. To the best of our knowledge we are the first to have looked at this in CHR-P patients.

We found a similar gradient structure as reported by other studies (23, 25, 28): One of the gradients span sensory and association areas and the other gradient span visual and sensorimotor areas. The order in which the gradients can be found is sometimes inconsistent across studies. Most often the sensory-to-association gradient is found to explain the most variance and the visual-to-sensorimotor gradient is found to explain the second most variance, but some studies found the gradient order to be switched (46, 54). This was also the case for our study.

For all groups we mainly found alterations in the visual-to-sensorimotor gradient opposed to the sensory-to-association gradient. The networks that showed the most alterations were the SMN for all patients and the VAN for CHR-P and ROP patients. In the sensory-to-association gradient, alterations were less pronounced and mainly shown by ROP patients again in the SMN and VAN. The pronunciation of alterations in the visual-to-sensorimotor gradient is supported by a recent study of gradients in schizophrenia (23) and furthermore in line with a study that reports altered visual-to sensorimotor rs-FC in psychosis (55). Moreover a previous study in the PRONIA sample found visual dysfunctions to be predicted by connectivity in the occipital network (14). Another study that focused on the sensory-to-association gradient, found similar patterns of alterations to our ROP group in the sensorimotor-to-visual gradient in schizophrenia, although differences were more widespread (28), which might be explainable by the progressed illness stage in their sample.

Our findings in the VAN are in line with previous studies of rs-FC, that showed aberrant connectivity of this network in psychosis and CHR-P patients (11, 13). As the effect for patients in general seems to be driven by the ROP and CHR-P patients, these alterations seem to be specific for the psychosis spectrum. As detection of salient stimuli or assignment of salience is disturbed in psychosis symptoms like delusions and hallucinations (56), it fits that disturbances are present in the VAN.

Alterations in the SMN that are shown by all patient groups imply the involvement of the SMN in transdiagnostic processes. The SMN has already been identified as a transdiagnostic region (57) and shows alterations across psychiatric disorders (16). Previous studies investigating functional brain gradients have also found alterations of basic networks in depression (25), as well as in psychosis (28). It has been suggested before that these results imply a “bottom-up dysregulation” (28), as alterations in the SMN can be found even when networks of higher functions remain intact (58).

Our findings of alterations in the SMN and the VAN are in line with the hypotheses of a disturbed integration of neural information in psychosis and support the theory of disturbed bottom-up information processing leading to impairment of higher functional networks.

We also found common alterations in ROP and its CHR-P state, with alterations being more pronounced in ROP patients than in CHR-P patients. These results are in line with the clinical staging model, which states that abnormalities in pathologic measures become more severe in later illness stages (59). It has also been shown that connectivity variance especially for the VAN, is associated with illness duration (60), further supporting our findings. In contrast to our results, a prior study did not find alterations in psychosis, but only in chronic schizophrenia, concluding that the hierarchical organization seems to change only with progression of illness (23). A reason for this may be their less impaired sample. Compared to their early psychosis sample, positive and negative symptom severity was higher in our ROP sample and even our CHR-P sample had a higher negative symptom score. Additionally, the size of our sample may have allowed us to detect subtle alterations, that might go undetected in a smaller sample.

### Functional differentiation

We found lower functional differentiation in ROD, manifesting in a lower range in the sensorimotor-to-visual gradient and lower within-network dispersion in the DAN and VAN. A lower range of gradient values in depression has been found before, but in the sensory-to-association gradient (25). However, the visual-to-sensorimotor gradient was not investigated in these studies.

In line with earlier studies (28), even though not significant after FDR correction, we found a trend of lower functional differentiation in psychosis. While regional and network analyses revealed similar alterations for psychosis and CHR-P patients, analysis of functional differentiation showed similarities between all patient groups in the form of lower between-network dispersion that resulted in an overall effect for patients. This is in line with findings of a study showing transdiagnostic de-differentiation in network organization in schizophrenia and depression (16). Regarding the range, there was also an effect for patient groups combined, driven by depression and CHR-P patients suggesting similarities between these groups. As CHR and ROD patients show similar clinical scores for functioning and depressive symptoms, it might not be surprising to find overlapping alterations. Especially as depression is in general the most prevalent comorbidity in individuals with CHR-P (61) and many CHR-P patients develop an affective disorder (62). Therefore, alterations in functional differentiation in CHR-P patients might reflect parts of the high-risk state other than the psychotic aspect. Additionally for CHR-P, with conversion rates lying around 20 to 30%, there is a high percentage of individuals who do not transition to psychosis (62–64), which could explain differences between CHR-P and psychosis patients.

### Association with clinical symptoms

We found associations between gradient scores of the DAN and VAN and clinical measures of functioning. It seems, that alterations in hierarchical brain organization are therefore more related to complex clinical measures rather than to single symptoms. Furthermore, we found associations of the VisDys score and within-network dispersion in the DAN and also with network values of the VAN in the sensorimotor-to-association gradient. This suggests that additionally to the FPN, that has been found to be predictive of VisDys in the PRONIA sample (14), disturbances in the VAN during hierarchical processing seem to be involved in VisDys symptoms. Additionally, within-network dispersion in the SMN was associated with cognition total and verbal learning, which might seem striking. Indeed alterations of the SMN have been related to cognitive dysfunction before (57). Nevertheless, correlations are rather small, which might be due to small effect sizes of our results regarding the hierarchical organization.

#### Association to receptor maps

We discovered a spatial association between multiple receptor maps and gradient abnormalities for ROD and CHR-P patients. Our findings for ROD patients are partly supported by previous studies who also found negative associations between case-control-differences and expression of 5Ht2a and 5Ht1b and positive associations with NET (65). Dopaminergic and serotonergic systems have been previously linked to mood disorder (66) and NET has been associated with functions in unimodal regions (67), which could explain its unique associations in the visual-to-sensorimotor gradient. That CHR-P patients show associations with many receptor systems might be due to the fact that this group is more heterogenous and often presents itself with different comorbidities. It also suggests that multiple neurotransmitter systems are involved in early disease processes. The overlap between receptor maps associated with the CHR-P state and ROD points into the same direction, highlighting again similarities between the ROD and CHR-P sample.

### Limitations

First, we used a parcellation-based approach in contrast to a vertex-based approach, which could have influenced the results regarding the gradients. Nevertheless, it has been shown before that for highly granular functional parcellation schemes such as the Schaefer parcellation, the correspondence to Mesulamś (22) scheme is high (46).

Second, in the context of multisite MRI data, the issue of site-effects represents a persistent challenge. We chose ComBat as a method to correct for site-effects, as it has often been used for MRI data. Nevertheless, this could potentially have had an effect on results as it is an artificial way of harmonizing data.

Third, our sample only includes early disease stages, which could explain the small effect sizes and weak associations to clinical symptoms. Even though our findings support the notion that alterations in psychosis might become more pronounced with progression of illness, our study is cross-sectional.

## Conclusion

In summary, we showed that the hierarchical organization of the brain is already disturbed in early disease stages, with the SMN and attentional networks exhibiting the greatest involvement. Our findings suggest that functional differentiation and gradient alterations of the SMN seem to be more general markers of psychiatric disease, while gradient alterations in the VAN seem to be more specific markers for the psychosis spectrum. In general, the findings point towards disturbed integration of bottom-up sensory input and attentional processes in psychiatric disorders. Our study suggests that alterations are already present in early disease stages and relate to functional outcomes and neurobiology. Future research should further investigate the role oft the SMN in transdiagnostic disease processes and examine the progression of disturbances in the hierarchical organization in a longitudinal sample.

## Figures and Tables

**Table 1:** Demographic characteristics of study groups

Characteristics	HC (*n*=376)	ROP (*n*=243)	ROD (*n=*212)	CHR-P (*n*=240)	Full sample (*n*=1071)	*F*(3,1067)/X^2^(3)	*p*
Sample size per site							
Munich	60^a,b^	87	75	85	307	5.95	.114
Milan	21	26	16	24	87	2.61	.456
Basel	56^a,b,c^	25	15	22	118	33.53	< .001
Cologne	64^a,b,c^	29	42	32	167	18.03	< .001
Birmingham	41^a,b,c^	14	16	16	87	22.84	< .001
Turku	51^b,c^	39^b^	20	27	137	16.31	< .001
Udine	72^a,b,c^	15	23	23	133	61.50	< .001
Münster	11	8	5	11	35	2.83	.419
Number of females	219^a^	102^b,c^	118	125	603	16.63	< .001
Age, mean (SD)	27.96 (6.34)^b^	27.89 (5.65)^b^	28.18 (6.41)^b^	26.03 (5.44)	27.56 (6.06)	6.73	< .001
Education Years, mean (SD)	15.65^a,b,c^ (2.70)	14.29 (7.82)	14.46^b^ (3.06)	13.49 (2.70)	14.62 (4.64)	11.75	< .001
CPZE, mean (SD)^1^	0 (0)^a,b,c^	153.00 (156.97)^b,c^	18.11 (66.21)	22.79 (55.75)	42.69 (102.82)	179.10	< .001

*Note*. CHR-P = clinical high-risk; HC = healthy controls; ROD = recent-onset depression; ROP = recent-onset psychosis.

Subscripts indicate significant differences after post-hoc t-test at the *p*FDR < .05 level after false discovery rate correction for multiple comparisons; ^a^ vs. ROP; ^b^ vs. CHR-P; ^c^ vs. ROD.

1 7 ROP patients with depot medication were excluded.

**Table 2:** Characteristics of participants included in analysis with clinical symptoms

Characteristics	ROP (*n*=234)	ROD (*n*=207)	CHR-P (*n*=236)	Full sample (*n*=677)	F(2,670)/X^2^(2)	*p*
Number of females	99^a^	114	122	334	7.87	.020
Age, mean (SD)	27.92 (5.67)^b^	28.34 (6.39)^b^	26.06 (5.40)	27.40 (5.89)	9.99	< .001
Education Years, mean (SD)	14.41 (7.96)	14.51 (3.07)	13.51 (2.71)	14.12 (5.24)	2.51	.082
BDI, mean (SD)	19.27 (10.47)^a,b^	24.51 (11.69)	24.42 (10.88)	22.67 (11.26)	17.05	< .001
PANSS Positive, mean (SD)	19.23 (5.86)^a,b^	8.53 (2.45)^b^	11.78 (3.59)	13.36 (6.17)	370.76	< .001
PANSS Negative, mean (SD)	15.65 (7.54)^a,b^	12.79 (5.27)	13.81 (6.67)	14.13 (6.70)	10.71	< .001
PANSS General, mean (SD)	34.61 (10.51)^a,b^	28.17 (7.59)	29.27 (8.13)	30.78 (9.30)	34.15	< .001
GF-S, mean (SD)	5.63 (1.53)^a,b^	6.34 (1.33)	6.17 (147)	6.04 (148)	14.95	< .001
GF-R, mean (SD)	5.10 (1.80)^a,b^	6.09 (178)	5.72 (176)	5.62 (1.82)	17.72	< .001
GAF-DI past month, mean (SD)	44.82 (13.31)^a,b^	55.24 (14.88)^b^	52.59 (13.06)	50.71 (14.40)	35.01	< .001
GAF-S past month, mean (SD)	40.94 (13.69)^a,b^	54.19 (12.74)	52.17 (11.08)	48.91 (13.82)	73.67	< .001
Cognition Total, mean (SD)	−0.67 (0.82)^a,b^	−0.16 (0.63)	−0.25 (0.59)	−0.37 (0.72)	34.85	< .001
WM, mean (SD)	−0.63 (0.99)^a,b^	−0.22 (0.84)	−0.14 (0.83)	−0.33 (0.92)	20.18	< .001
Social Cognition, mean (SD)	−0.41 (1.14)^a,b^	−0.08 (0.90)	−0.19 (0.90)	−0.23 (1.00)	6.76	.001
Verbal Learning, mean (SD)	−0.50 (1.13)^a,b^	−0.10 (0.92)	−0.17 (0.95)	−0.26 (1.02)	10.30	<.001
SoP, mean (SD)	−0.80 (0.88)^a,b^	−0.23 (072)	−0.35 (0.66)	−0.47 (0.80)	36.08	< .001
Attention, mean (SD)	−0.98 (1.96)^a,b^	−0.15 (1.68)	−0.42 (170)	−0.53 (1.82)	12.51	< .001
VisDys, mean (SD)	3.96 (7.92)^a^	0.71 (2.03)^b^	3.81 (6.16)	2.91 (6.18)	20.04	< .001

*Note*. BDI = Beckś Depression Inventory; CHR-P = clinical high-risk; CPZE = chlorpromazine equivalent; GAF-DI = Global Assessment of Functioning Disabilities; GAF-S = Global Assessment of Functioning Symptoms; GF-R = Global Functioning Role; GF-S = Global Functioning Social; HC = healthy controls; ROD = recent-onset depression; ROP = recent-onset psychosis; SoP = speed of processing; VisDys = visual dysfunctions; WM = working memory.

Subscripts indicate significant differences after post-hoc t-test at the *p*FDR < .05 level after false discovery rate correction for multiple comparisons; ^a^ vs. ROD. ^b^ vs. CHR-P.
